# Spectroscopically clean Au nanoparticles for catalytic decomposition of hydrogen peroxide

**DOI:** 10.1038/s41598-021-89235-y

**Published:** 2021-05-06

**Authors:** Kai Liu, Shuyue He, Lin Li, Yi Liu, Zhihua Huang, Tinghan Liu, Han Wu, Xiaolin Jiang, Kun Liu, Fei Tian

**Affiliations:** 1 National Innovation Center of Advanced Rail Transit Equipment, Zhuzhou, 412000 Hunan China; 2 Department of Chemical Engineering and Materials Science, Stevens Institute of Technology, Hoboken, NJ 07030 USA

**Keywords:** Nanoscale materials, Catalyst synthesis, Raman spectroscopy

## Abstract

Au nanoparticles synthesized from colloidal techniques have the capability in many applications such as catalysis and sensing. Au nanoparticles function as both catalyst and highly sensitive SERS probe can be employed for sustainable and green catalytic process. However, capping ligands that are necessary to stabilize nanoparticles during synthesis are negative for catalytic activity. In this work, a simple effective mild thermal treatment to remove capping ligands meanwhile preserving the high SERS sensitivity of Au nanoparticles is reported. We show that under the optimal treatment conditions (250 °C for 2 h), 50 nm Au nanoparticles surfaces are free from any capping molecules. The catalytic activity of treated Au nanoparticles is studied through H_2_O_2_ decomposition, which proves that the treatment is favorable for catalytic performance improvement. A reaction intermediate during H_2_O_2_ decomposition is observed and identified.

## Introduction

Gold nanoparticles (Au NPs) with different sizes^1,2^, shapes^3^, and compositions^4,5^ have been well studied using aqueous or organic solution synthesis in catalytic applications. Development of Au NPs with improved catalytic activities and selectivity on chemical reactions is very appealing since it allows accurate studies on the structure–activity relationships and reaction mechanisms^6,7^. Au NPs are also one of the most investigated plasmonic nanostructures, due to their localized surface plasmon resonance (LSPR) in the visible wavelength range. LSPR arises from collective oscillation of electrons in the conduction band of nanostructured metal when irradiated by light^8,9^. The resultant electromagnetic field enhancement has been a key enabling feature for a multitude of applications, with sensing and detection using surface-enhanced Raman spectroscopic (SERS) being a prime example^10^. Sensitivities on the order of 10^–9^ can be routinely achieved^11^. Single molecule detection has also been reported^11–14^. Au NPs with dual functionalities work as the catalyst and highly sensitive SERS probe offer exciting opportunities to study sustainable and green catalytic processes.

Several efforts have been made in the SERS study on catalytic process using colloidal Au NPs. For example, Joseph et al. reported a study on the kinetics of gold and platinum mixed nanoparticle catalyzed reduction of *p*-nitrothiophenol (PNTP) to *p*-aminothiophenol (PATP) with sodium borohydride using SERS^15^. In situ studies were carried out by Li et al. to monitor the intermediated (AuOH/AuO) species during electrooxidation processes using shell-isolated Au NPs-enhanced Raman spectroscopy^16^. However, colloidal Au NPs are usually capped with specific surfactant or ligands, which enable particle size, shape and stability control during synthesis. While these capping ligands are born with nanoparticle synthesis, they are detrimental for catalytic application since they block the binding sites where the reaction occurs^17^. Also, the capping ligands introduce major uncertainty when the capped nanoparticles are used as surface-mediated functional materials such as catalysts, whose performance critically depends on the surface composition/structure^18,19^. Therefore, it is critical to establish a proper experimental protocol for ligands removal and improved catalytic activity, meanwhile retaining the particles sensitivity in chemical sensing.

However, the removal of these capping ligands is problematic, this issue has been well recognized but more or less unresolved in the catalysis investigation over colloidal nanoparticles with capping ligands due to its complexity^20^. Some methods have been reported to remove the capping ligands and increase the catalytic activity of nanoparticles, such as solvent extraction^21^, fast extreme high temperature annealing^22^, ozone treatment^23^ and chemical treatment^20^. All these methods can remove those capping ligands and activate catalysts to some extent. But those methods either still leave residues or require expensive equipment to be effective. Since the Au NPs size need be above 10 nm to be effective as SERS probes, thermal treatment at mild conditions by control the temperature and time can potentially work as an efficient means to remove ligands and preserving SERS sensitivity without changing the morphology of particles due to the melting-point depression^24,25^. In this work, we studied extensively the effect of treatment temperature and time on the morphology and SERS sensitivity of colloidal synthesized 50 nm Au NPs. We show that the catalytic activity of Au NPs under the optimal treatment conditions increased enormously compared with untreated counterparts. In situ studies were performed on the treated Au NPs to study the intermediates during particle-catalyzed H_2_O_2_ decomposition.

## Methods

### Preparation of monodisperse Au nanoparticles

Monodisperse 50 nm Au particles were supported on a Si wafer with a SiO_2_ oxide surface layer (5 × 5 mm diced Si wafer, type <100>, Ted Pella, Redding, California) as described in our previous work^1^. The SiO_2_ surface was pre-covered by poly(allylamine hydrochloride) (PAH) polymer (weight-average molecular weight 15,000 g mol^−1^, Sigma-Aldrich) in order to minimize agglomeration of metal nanoparticles. PAH was first dissolved in Milli-Q water, purified water that was filtered with Barnstead ion-exchange columns and further purified by passing through Millipore (Milli-Q) columns. The pH of the PAH aqueous solution was adjusted to 9 using sodium hydroxide (0.1 M NaOH standard solution, volumetric, Sigma-Aldrich). The SiO_2_ surface was exposed to the PAH aqueous solution for 30 min, then rinsed three times with Milli-Q water to remove any free or loosely bound PAH molecules.

Monodisperse 50 nm Au particles with stabilizing citrate organic ligands in colloidal solutions were used in the preparation of Au/SiO_2_^1^. The 50 nm Au particles colloidal solutions (NanoXact, citrate, 2 mM in sodium citrate solution, Nanocomposix, San Diego, California) had a concentration of 0.05 mg of Au ml^−1^. The Au nanoparticles were deposited onto the PAH-coated SiO_2_ surface by adding drop-wise 20 μl of a colloidal solution, allowing it to dry and then rinsing three times with Milli-Q water to remove any free or loosely bound Au nanoparticles. The number of deposited Au nanoparticles was adjusted in such a way that the ratio of the surface areas for Au nanoparticles and the SiO_2_ support remained constant at ~ 0.13 m^2^ Au/m^2^ SiO_2_. The surface area of Au was estimated by assuming that the Au nanoparticles were spherical. The surface coverage density for 50 nm Au particles was 14.2 ± 0.2 particles per SiO_2_ μm^2^. Most Au nanoparticles were isolated, only a small fraction formed larger particles as dimers and trimers.

After the rinsing and drying procedure at ambient conditions, the Au/SiO_2_ samples were exposed to a thermal treatment in order to remove the citrate organic ligands and, thus, produce clean metallic Au nanoparticles. The Au/SiO_2_ samples were treated at an appropriate temperature (150, 250 and 350 °C) in a box furnace (Thermo Scientific) under N_2_ (5.0 ultrahigh purity, 99.999%, Praxair) flow for varying time period (0.5, 1, 2 and 3.5 h). After the appropriate time interval, the sample was quickly remove from the furnace and left cooling in air. Morphology changes due to the thermal treatment were evaluated by analysing images obtained with a scanning electron microscope (SEM, Zeiss Auriga) at 3 kV with a working distance of 5.5 mm. The SEM image analysis was performed using ImageJ software from the National Institutes of Health.

### H_2_O_2_ decomposition kinetic measurements

Kinetic measurements for H_2_O_2_ decomposition with the formation of gas-phase O_2_ and liquid-phase H_2_O were performed following the same method in our previous work^1^ at ambient conditions (room temperature, atmospheric pressure, open to air) in a custom-made open cylindrical glass Raman spectroscopic cell, which served as a batch reactor. The cylindrical cell was made using a glass tube (10 mm outer diameter, 10 mm height, non-sterile, borosilicate glass, Bellco Glass, Inc., Vineland, New Jersey) that was adhesively bonded at one end to a glass slide (22 × 22 mm surface, 0.15 mm thickness, coverglass, Electron Microscopy Sciences, Hatfield, Pennsylvania) using molten wax (quickstick 135 mounting wax, Electron Microscopy Sciences, Hatfield, Pennsylvania). The pH of the initial 30 wt% H_2_O_2_ solution in water (Fisher Scientific) was adjusted to 9 by diluting to 15 wt% with sodium hydroxide (0.1 M NaOH, volumetric, Sigma-Aldrich). In each experiment, 100 μl of a pH-adjusted 15 wt% H_2_O_2_ solution was used for monitoring decomposition reactions with Raman spectroscopic measurements. In parallel experiments using the same Raman spectroscopic cells, the reaction rate for H_2_O_2_ decomposition was determined by analyzing the H_2_O_2_ conversion as a function of reaction time with volumetric titration using potassium permanganate (KMnO_4_, 99+%, ACS reagent, Fisher Scientific)^1^. Each H_2_O_2_ conversion measurement was obtained in a separate experiment using 100 μl of the pH-adjusted 15 wt% H_2_O_2_ by varying the reaction time because only one titration could be performed with this limited amount of the H_2_O_2_ solution.

### Raman spectroscopic measurements

Raman spectroscopic measurements were performed using a custom-built setup. The setup and its capabilities were described previously^1^. Briefly, a 632.8 nm wavelength laser beam was spatially filtered and reflected from a Chroma Z633RDC dichroic mirror, and excited the back aperture of a Thorlabs 20 × 0.55 N.A. objective. The excitation laser intensity in front of the objective was ~ 8 mW. The sample cell was placed on a Newport ULTRAlign 561D translation stage. The Raman signal from the objective passed through the dichroic mirror. A collimator was used to focus the signal into a spectroscopic grade multimode fibre (400 μm core, Newport). A fibre-coupled Acton SpectraPro 2300 spectrometer with a Roper Scientific liquid nitrogen cooled CCD detector was used in spectrum acquisition. The objective was used to produce a large laser-focusing beam (~ 1 mm in diameter). These large excitation spots as well as the uniform distribution of the nanoparticles on the surface allowed us to obtain a stable signal from adsorbates on the surface of Au nanoparticles. A short exposure time of 2 s was chosen in order to better detect reaction intermediates. All Raman measurements were performed using an acquisition time of 20 s (exposure time of 2 s × 10 integral exposures). During H_2_O_2_ decomposition in the presence of Au, the laser focus was adjusted to be on the surface of the Au nanoparticles.

## Results and discussion

**Figure 1:**
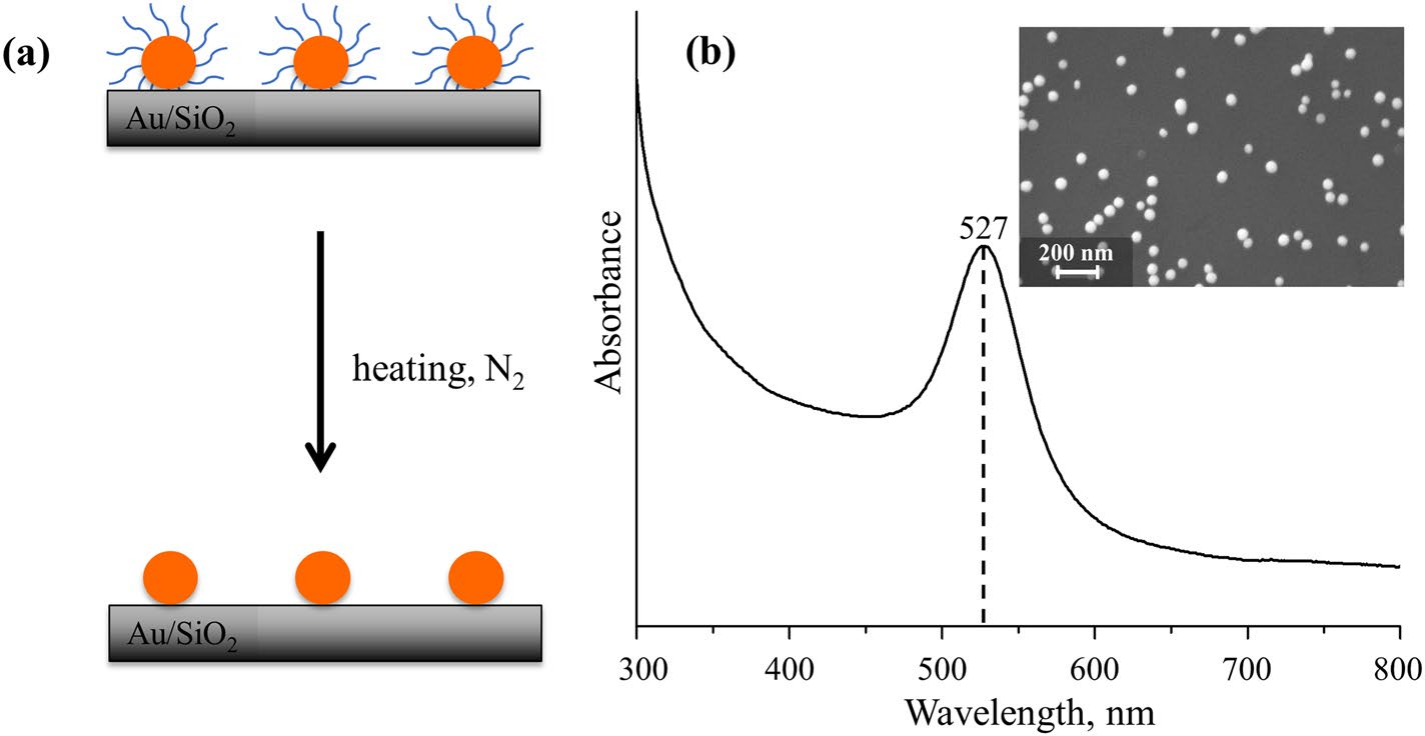
(**a**) Schematic of ligands removal of 50 nm Au NPs supported on SiO_2_ using mild thermal treatment. (**b**) UV–Vis absorption spectrum of colloidal 50 nm Au NPs. The inset in (**b**) is a SEM micrograph of monodisperse Au NPs on SiO_2_.

The study consists of a comprehensive exploration about effects from thermal temperature and treatment time on the Au NPs SERS sensitivity and resultant catalytic activity. It is worth to mention that stabilizing ligands such as citrates are intrinsic and necessary during Au NPs synthesis. However, the presence of citrate on Au NPs surface will block the adsorption of reactants and reaction intermediate, thus interferes catalytic activity and decreases the detection sensitivity. As shown in Fig. 1a, the essence for this thermal treatment to supported Au NPs is efficiently removal of organic ligands that indispensible in the NPs synthesis, while keeping the NPs SERS sensitivity effectively. Figure 1b presents the UV–Vis spectrum of 50 nm Au NPs, which clearly displays an evident absorption feature at around 527 nm. The inset in Fig. 1b shows the SEM micrograph of Au NPs, indicating the well-dispersed NPs on SiO_2_.

**Figure 2:**
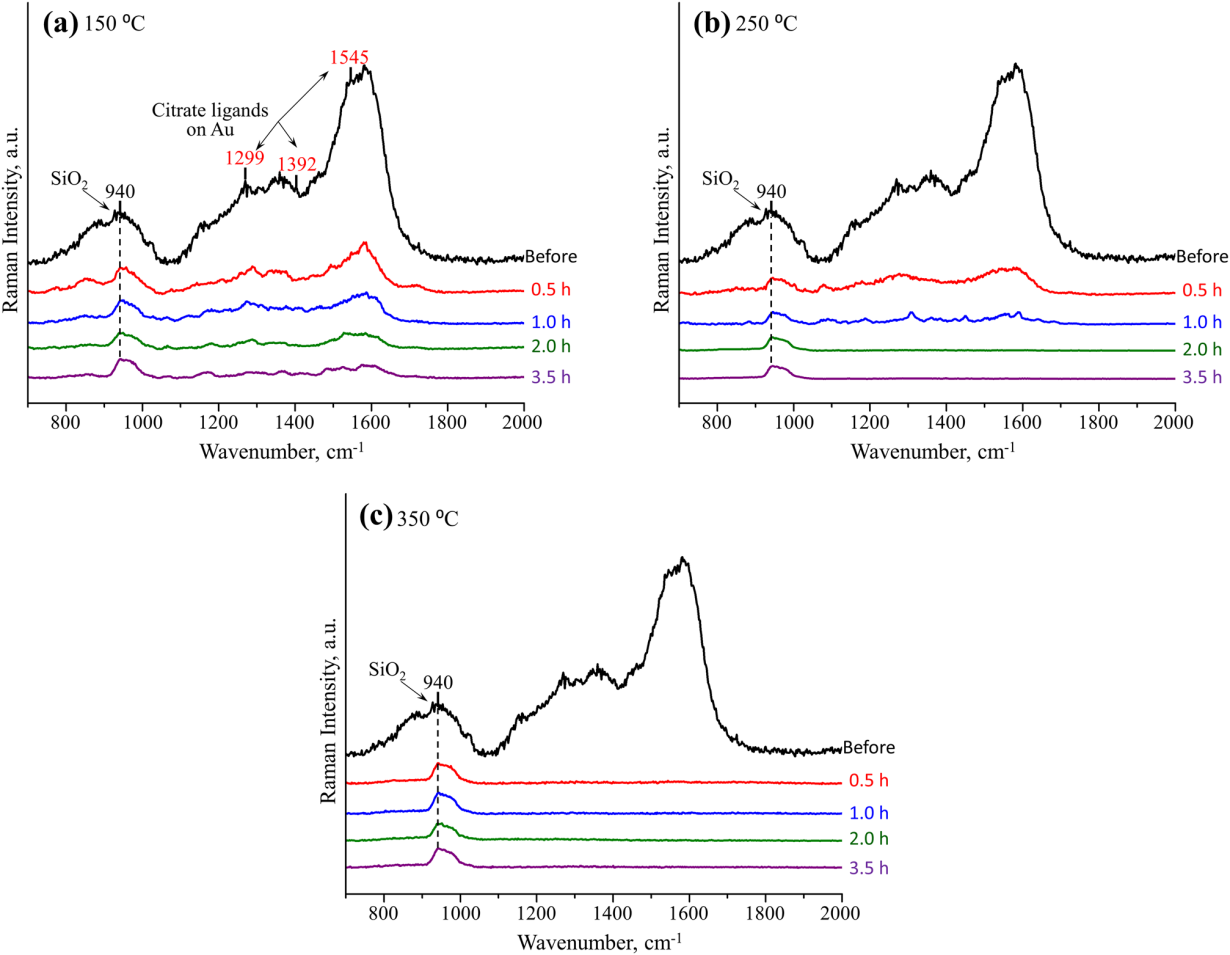
Raman spectra of 50 nm Au NPs supported on SiO_2_ collected after thermal treatment at (**a**) 150, (**b**) 250 and (**c**) 350 °C for varying time period (0.5, 1, 2, 3.5 h) under N_2_ atmosphere. Spectra were acquired at 632.8 nm (~ 8 mW, 20 s).

In order to figure out what thermal temperature and treatment time can be utilized to effectively remove the organic capping ligands, the Au/SiO_2_ samples were first treated at an appropriate temperature (150, 250 and 350 °C) under N_2_ flow for varying time period (0.5, 1, 2 and 3.5 h). Raman spectroscopy was employed to evaluate the degree of removal of organic ligands by looking at the features coming from sodium citrate. The presence of surface-attached citrate on the as-synthesized Au NPs is evidenced by the rich Raman signature bands at around 1299, 1392, 1545 cm^−1^ for C–H bending frequency of citrate^26^, as shown in Fig. 2. At 150 °C, the Raman intensity of capping citrate decreased gradually with treating time but still show rich features even after 3.5 h in Fig. 2a. By treating the NPs at 250 °C, the Raman background associated with citrate showed the same trend until totally disappeared after 2 h, indicating complete removal of the capping citrate and thus fully availability of active sites for surface adsorption as illustrated in Fig. 2b. The only peak at ~ 940 cm^−1^ represent the signature bands from the SiO_2_ substrate, which is identical with the Raman spectrum of the substrate alone. Further increasing the treatment time for 3.5 h still resulted in clean Raman background of Au/SiO_2_. In Fig. 2c, the removal of capping citrate was completed in the even shorter period of only 0.5 h at 350 °C. It was obvious that complete removal of capping citrate can be realized either at 250 °C for at least 2 h or 350 °C for 0.5 h and above.

**Figure 3:**
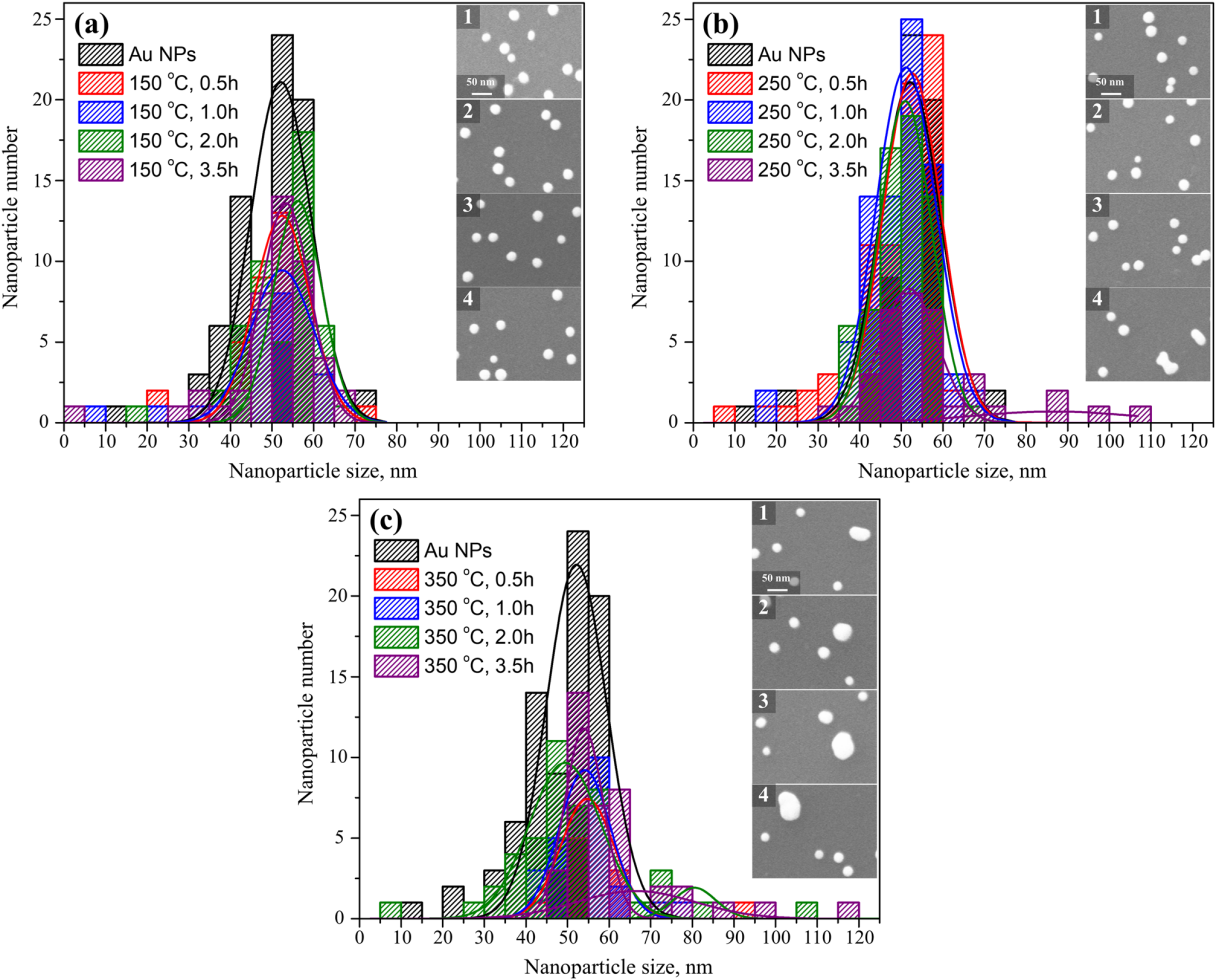
Histograms of particle size distributions for the initial Au NPs and the treated Au NPs after thermal treatment at (**a**) 150, (**b**) 250 and (**c**) 350 °C for varying time period. Lines represent Gaussian fittings of the distributions. The insets are the SEM micrographs of Au NPs on SiO_2_ after each temperature treatment for 0.5, 1, 2, 3.5 h (From top to bottom).

Size distribution histograms of the initial supported Au NPs and of the treated NPs at different thermal conditions are reported in Fig. 3. After removal of the capping ligands at 150 °C, the distributions of the treated Au NPs size were as narrow as the initial NPs size distribution as can be seen from Fig. 3a. There is virtually no change in the overall morphology, including particle size and distribution, for Au NPs on SiO_2_ after thermal treatment at 150 °C, compared to the untreated counterpart, as illustrated in the inset SEM images in Fig. 3a(1–4). By treating the NPs at 250 °C, the distributions of the treated Au NPs were still as narrow as the initial NPs until 2 h. However, a second Gaussian distribution peak around 85 nm emerged after 3.5 h thermal treatment at 250 °C, indicating a substantial coalescence of the neighboring particles, which is more visible from inset SEM image in Fig. 3b(4). After thermal treatment at 350 °C, the Au NPs in close proximity fuse to form single, large particles began for only 0.5 h. Particle enlargements intensified with increased treatment time, resulting in increased average particle size and reduced particle coverage density as illustrated in Fig. 3c(1–4). The morphological changes of Au NPs on SiO_2_ treated at 350 °C are consistent with the size distribution histograms, which clearly showed size larger than 50 nm and second Gaussian distribution. For the purpose of preserving the original morphological stability, the thermal treatment temperature and time should be limited below 250 °C and 2 h respectively. Therefore, based on above results, the preferable strategy to efficiently remove the capping ligand on Au NPs supported on SiO_2_ meanwhile maintaining the morphological stability is thermally treated the samples at 250 °C for 2 h.

**Figure 4:**
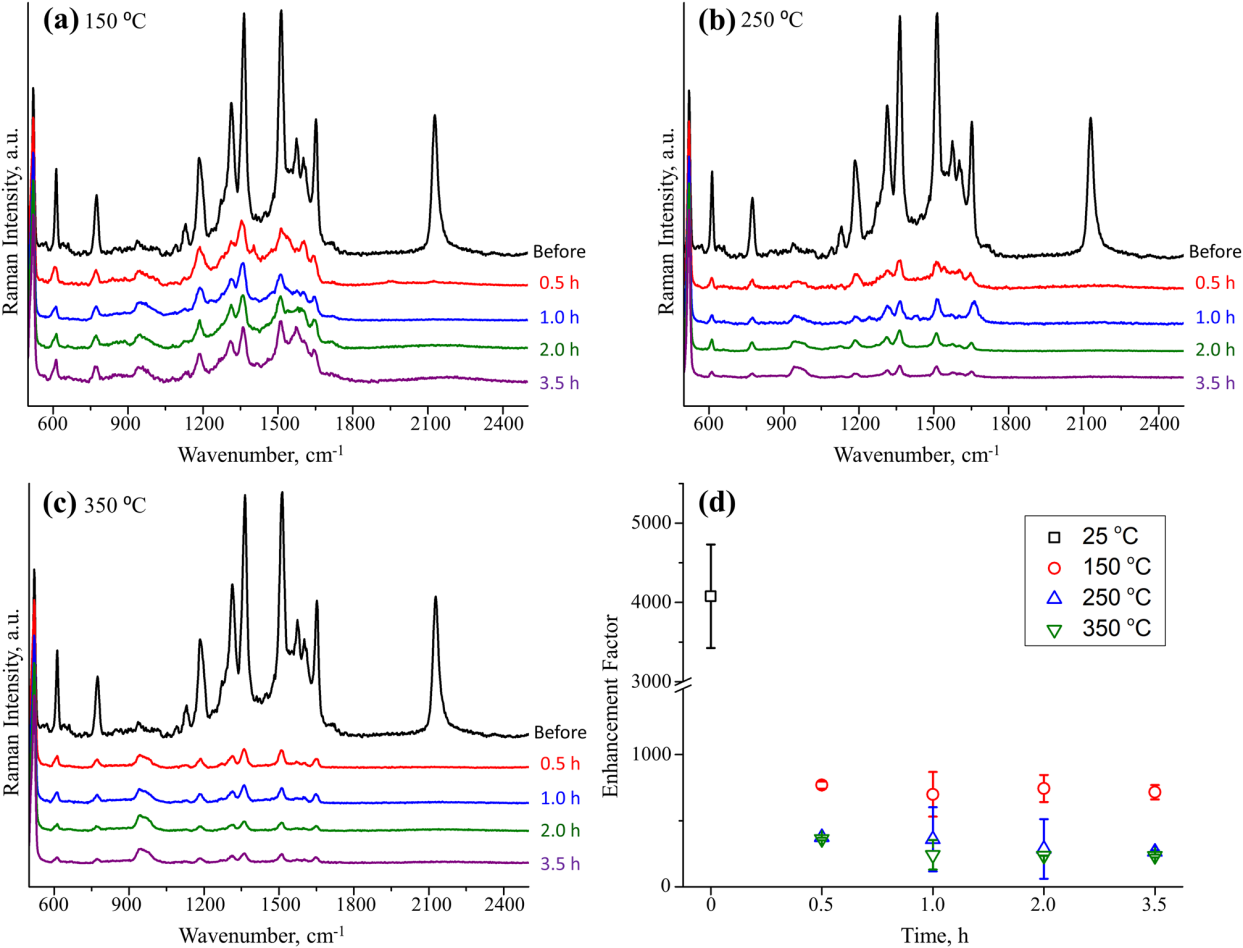
Raman spectra of R6G on 50 nm Au NPs supported on SiO_2_ collected after thermal treatment at (**a**) 150, (**b**) 250 and (**c**) 350 °C for varying time period (0.5, 1, 2, 3.5 h) under N_2_ atmosphere. Spectra were acquired at 632.8 nm (~ 8 mW, 20 s). (**d**) Summary of calculated EFs for R6G using the band at 1364 cm^−1^ for Au NPs supported on SiO_2_ after thermal treatment.

The SERS enhancement of colloidal nanostructures has a strong dependence on the materials, shape, size, surface chemistry and even the inter-structure gap^33^. Table 1 presents selected SERS studies of plasmonic nanostructures. Enhancement factor (EF), an important indicator of SERS sensitivity, was calculated using the equation: EF = (*I*_*S*_/*C*_*S*_)/(*I*_*N*_/*C*_*N*_), where *I*_*S*_ and *I*_*N*_ are integrated intensities of a characteristic band from SERS and normal Raman, and *C*_*S*_ and *C*_*N*_ are concentrations of model analyte used in SERS and normal Raman experiments, respectively. As we can seen from Table 1, generally, Au has less SERS enhancement than Ag nanostructures and smaller inter-structure gap gives higher enhancement than monomers. To evaluate the effect from thermal temperature and time on SERS sensitivity of the Au NPs, R6G was used as a model analyte in the ambient-temperature SERS measurements on 50 nm Au NPs supported on SiO_2_ before and after the various heat treatments process. The SERS spectra of 10^–5^ M R6G obtained from 50 nm Au NPs/SiO_2_ before and after thermal treatment are shown in Fig. 4a–c. Note that at all thermal treatment temperature, the Raman intensity underwent a striking and rapid reduction upon annealing after only 0.5 h, as compared to unannealed samples. Also, further increasing the temperature will further decrease the SERS sensitivity. Figure 4d shows the analytical EFs calculated using the band at 1364 cm^−1^ of R6G as a function of time at different treatment temperature. According to the results, while there was a relatively minor difference as time increased at 150 °C, the EF of Au NPs experienced a downward tread when time increased from 0.5 to 3.5 h at 250 °C and 350 °C. The Au NPs samples treated at 250 °C for 2 h still preserving enormous SERS enhancement that is perquisite for chemical sensing applications.

**Table 1:** Summary of reported SERS enhancement of colloidal nanostructures with different materials, shape, size, surface chemistry and inter-structure gap.

Materials	Shape	Size	Model analyte	Concentration	EF
Au/Pt^15^	Particles	Au: 40 nm/Pt: 2 nm in diameter	Crystal violet	5 × 10^–6^ M	10^6^
Ag^27^	Clusters	Wirelike Ag cluster with interpartile gap of 1.7 nm	Methylene blue	1 × 10^–12^ M	10^8~^10^10^
Au^28^	Rings	Outer diameter: 40 nm Inner diameter: 30 nm	Trans-1,2-bis(4-pyridyl)ethylene	2 ppm	8.85 × 10^6^
Au^29^	Stars	70 ± 5 nm in diameter	Single texas red dye	1.5 × 10^–3^ M	3 × 10^9^
Au^30^	Triangles	Edge 103.6 ± 4.2 nm Thickness 62.0 ± 4.3 nm	4MBA	2.5 × 10^–4^ M	10^4^
Au^31^	Sphere	40 nm in diameter	Triarylmethane dye	5 × 10^−6^ M	2 × 10^3^
Ag^32^	Particles	15.6 nm in diameter	Rhodmine 6G	10^−6^ M	5 × 10^5^
This work	Particles	50 nm in diameter	Rhodmine 6G	10^–5^ M	4 × 10^3^

Au NPs immoblized on SiO_2_ are mostly discretely distributed as monomers, there exists a significantly portion of dimers and even trimmers in direct contact or separated by nm gap shown in Fig. 1. These clusters are known to form hot spots that give rise to very high SERS sensitivities^9^. When the Au NPs were treated at 150 °C and due to high surface atom mobility, necking began to take place, which leading to reduction in hot spots and thus the SERS sensitivity. This process is responsible for the rapid decrease in the EFs although thermal desorption of capping ligands has the benefit of clean SERS background as well as increased binding sites for analytes. The difference of Au NPs EF as the treatment time increased at 150 °C is neglectable due to the trade-off of decreased SERS background and continued necking. At 250 °C, necking continued for Au NPs and led to further reduction in the hot spots and continued decline in EF. The downward trend as the time increased results from the continued necking and even fusion into larger individual peanut shape particles. When Au NPs treated at 350 °C, fusion into larger spherical particles began for just 0.5 h and the coalescence accelerated as treatment time increased, which leads to the formation of Au NPs as larger as 120 nm in diameter. The substantial increase in particle size greatly reduces the SERS activity. The adverse effect of size increase counters the beneficial effect of free metal surface, yielding a relatively smaller EF compared to 150 °C and 250 °C. The removal of the capping ligands on Au NPs surface at 250 °C for 2 h not only maintain the narrow size distribution of the particles, their shape and composition but also the SERS sensitivity which can be utilized for in-situ chemical sensing in catalytic applications.

It is indeed important to keep in mind that, despite Raman suggests the removal of ligands has taken place, the catalytic activity is the final proof that the method is applicable. As shown in Table 2, some reported methods have been utilized to remove the capping ligands and increase the catalytic activity of nanostuctures to some extend. However, to the best of our knowledge, the reported catalysis has size less than 10 nm and can not be used as SERS probe for in-situ chemical sensing.

**Table 2:** Summary of reported capping ligands removal method on the catalytic activity of nanostructures.

Catalyst	Size	Modification method	Catalytic reaction	Effects
Au/TiO_2_^21^	5.1 nm	Calcination (200 °C, N_2_)	CO oxidation	Non-treated: 0% conversion Treated: 8% conversion
Pd/CeO_2_^22^	5.5 nm	Fast thermal annealing (700 °C, 30 s)	CO oxidation	Non-treated: 1% conversion Treated: 8% conversion
Au nanoclusters^34^	1.6 nm	“Soft” nitriding	Electrocatalytic methanol oxidation	Non-treated: 0.006 cm/s electrochemical rate constant Treated: 0.02 cm/s electrochemical rate constant
Pt nanoparticles^35^	2.8 nm	Low-temperature thermal annealing (185 °C, 5 h)	Electrocatalysts oxygen reduction reaction	CO–Pt interaction was examined after treatment
Au/TiO_2_^23^	1.2 nm	Ozone exposure	CO oxidation	Treated: higher activity, 50% conversion at 335 °C
This work	50 nm	Thermal treatment (250 °C, 2 h)	H_2_O_2_ decomposition after 3 h	Non-treated: 47.6% Treated: 82%

**Figure 5:**
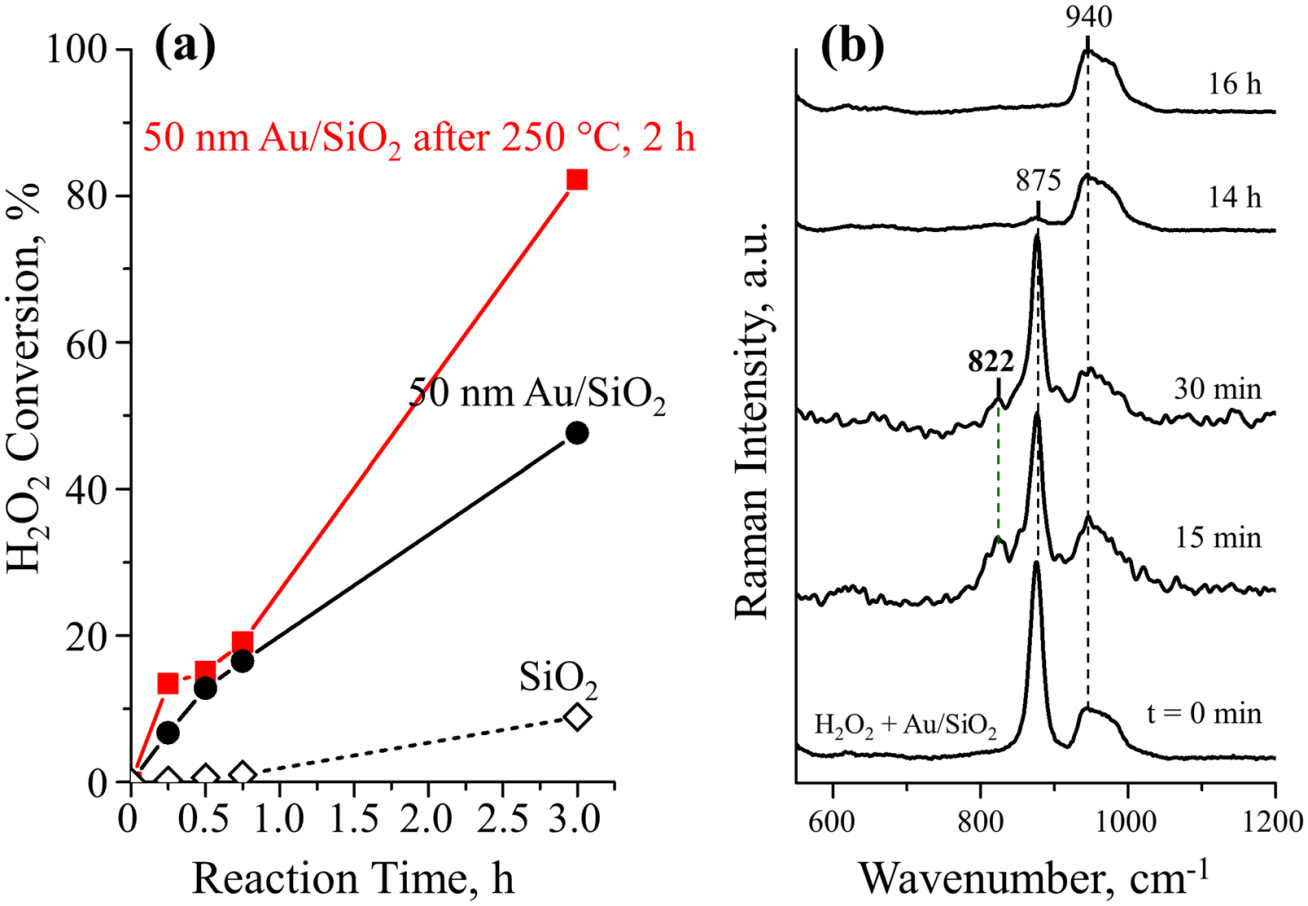
H_2_O_2_ decomposition over 50 nm Au/SiO_2_. (**a**) Dependence of decomposition rates on the Au NPs with and without thermal treatment at 250 °C for 2 h. (**b**) Evolution of in situ Raman spectra as a function of reaction time plus a comparative spectrum for H_2_O_2_ over SiO_2_ showed the presence of reaction intermediates with band at 822 cm^−1^.

We therefore studied the effects of thermal treatment method on Au NPs catalyzed H_2_O_2_ decomposition reaction. 50 nm Au NPs supported on SiO_2_ which act as both catalyst and SERS probe after thermal treatment at 250 °C for 2 h were employed for the in situ monitoring of H_2_O_2_ decomposition. The H_2_O_2_ conversion of Au NPs supported on SiO_2_ before and after thermal treatment provide qualitative results of the effectiveness of the capping ligand removal as shown in Fig. 5a. It was found that while the untreated Au NPs supported on SiO_2_ gave 47.6%, the H_2_O_2_ conversion at pH 9 on thermal treated Au reach 82%, which was almost double compared to the untreated counterpart. When a blank experiment was conducted in the absence of Au, the rate of H_2_O_2_ decomposition was dramatically lower over the SiO_2_ support: only 8% conversion after 3 h. This confirmed that thermal treatment at 250 °C for 2 h can increase the catalytic activity of Au by removing the organic citrate ligands and producing clean surface.

Time evolution of in situ Raman spectra of H_2_O_2_ decomposition on treated 50 nm Au NPs at selected time intervals has been presented in Fig. 5b. As soon as H_2_O_2_ with pH 9 was added, the spectrum exhibited a characteristic band at 875 cm^−1^, which can be attributed to *ν*(O–O) stretching vibrations of H_2_O_2_^36,37^. After 15 min, along with the formation of gas-phase O_2_ as the decomposition product, a new band emerged at 822 cm^−1^. This new band was consistently presented after 30 min, with intensities decreasing as time increased. After 14 h, while the new band at 822 cm^−1^ disappeared, the intensity of band at 875 cm^−1^ also decreased a lot. The H_2_O_2_ feature at 875 cm^−1^ finally vanished after 16 h, indicating the total conversion of the H_2_O_2_ into H_2_O. Since the new band at 822 cm^−1^ was observed only during the H_2_O_2_ decomposition, it must be due to reaction intermediate.

The band at 822 cm^−1^ in Fig. 5b is proved to be the characteristic of the *ν*(O–O) stretching vibration in hydroperoxy (Au-OOH) species on Au. The same result has been observed when H_2_O_2_ decomposed in 400 nm Au/SiO_2_ treated under the same conditions^1^. Also, it falls within the range reported for end-on transition metal-bound hydroperoxide ligands. For instance, Yeo and coworkers^38^ observed that the OOH intermediate from oxygen evolution reaction on Au lies between 815 and 830 cm^−1^. Rajani et al. identified *ν*(O–O) vibration located between 824 and 830 cm^−1^ for Co-OOH complexes^39^ and Liu et al. assigned the band at 796–810 cm^−1^ to *ν*(O–O) vibration of Fe-OOH complexes^40^. Also, based on the theoretical simulation, *ν*(O–O) of H_2_O_2_ was calculated to be 874 cm^−1^ ^41^ and *ν*(O–O) of OOH was determined to be in the range from 831 cm^−1^ for small Au clusters to 806 cm^−1^ for Au(111)^1^, in close agreement with our experimental results. The likelihood that the 822 cm^−1^ band originates from Au-OH complex can be excluded. The deuterium isotopic substitution measurement by Li^16^ revealed that the bending frequency of Au-OH will downshift from 790 cm^−1^ to low wavenumber 694 cm^−1^ in deuterated water, far different from the observed 822 cm^−1^ peak in our measurement. Also, the Au-OH stretching frequency is a broad band at ~ 360 to 420 cm^−1^^16^. Based on above analysis, the band at 822 cm^−1^ in Fig. 5b can be attributed to the *ν*(O–O) stretching vibration from hydroperoxy (Au-OOH) species on Au. Therefore, effective removal of capping ligands on Au by thermal treatment method at 250 °C for 2 h can not only increase the Au catalytic activity but also maintain the high sensitivity of SERS performance thus in situ studying the reaction intermediates in multiple and diverse areas of science and technology.

## Conclusions

In summary, a simple thermal treatment procedure at 250 °C for 2 h was developed for producing spectroscopically clean monodisperse 50 nm Au nanoparticles, which has been proved in the Raman spectra of Au on SiO_2_. This procedure allowed the morphological stability and SERS sensitivity of Au nanoparticles been preserved. The kinetic measurements for H_2_O_2_ decomposition on treated Au showed that the reaction rate increased significantly compared to untreated Au counterparts. Using treated Au nanoparticles work as both Raman probe and catalyst, a reaction intermediate OOH with band at 822 cm^−1^ during H_2_O_2_ decomposition was observed with spectroscopic measurement. We hope this simple thermal treatment method can be applied to the development of improved Au-based catalysts, biomedical and optical sensor, batteries and in other areas.
